# Deltamethrin interacts with *Culex quinquefasciatus* odorant-binding protein: a novel potential resistance mechanism

**DOI:** 10.1186/s13071-021-05041-5

**Published:** 2022-01-03

**Authors:** Rui-xin Shen, Yi-ting Wang, Jia-hong Wu, Ning Zhang, Heng-duan Zhang, Dan Xing, Yan Chen, Chun-xiao Li, Tong-yan Zhao

**Affiliations:** 1 State Key Laboratory of Pathogen and Biosecurity, Beijing Key Laboratory of Vector Borne and Natural Focus Infectious Disease, Beijing Institute of Microbiology and Epidemiology, Beijing, 100071 China; 2 Guizhou Medical University, Guiyang, 550000 China

**Keywords:** *Culex quinquefasciatus*, Deltamethrin, Resistance, Odorant-binding protein 28, RNA interference

## Abstract

**Background:**

Odorant-binding proteins (OBPs) play important roles in many physiological processes of mosquitoes. Previous high-throughput sequencing studies have revealed that some OBPs of *Culex quinquefasciatus* might be involved in the development of resistance to insecticides.

**Methods:**

Based on the results of sequencing analyses, the* OBP28* gene was selected for evaluation in this study. Three laboratory strains of *Cx. quinquefasciatus* [susceptible strain (SS), deltamethrin-resistant strain 1 (HN) and deltamethrin-resistant strain 2 (RR)] were first examined by using the Centers for Disease Control and Prevention bottle bioassay, after which the expression level of the* OBP28* gene in the susceptible and deltamethrin-resistant strains was determined by real-time quantitative polymerase chain reaction. The *OBP28* gene in deltamethrin-resistant strain RR was silenced using RNA interference technology. The expression level of *OBP28* and the resistance level were tested in the silenced strain and control strain after microinjection of double-stranded RNA for a 48-h interference period. Four field-collected strains (henceforth ‘field strains’) of *Cx. quinquefasciatus* were also examined for their resistance to deltamethrin and levels of OBP28 expression. Finally, a correlation analysis between deltamethrin resistance and gene expression was carried out for all seven strains, i.e. the four field strains and the three laboratory strains.

**Results:**

In the bioassay, the mortality of SS, HN and RR was 100%, 21.33% and 1.67%, respectively. The relative expression levels of *OBP28* in strains HN and RR were 6.30- and 6.86-fold higher, respectively, than that of strain SS. After silencing of the *OBP28* gene, the mortality of strain RR was 72.20% and that of the control strain 26.32%. The mortality of strain RR increased significantly after interference compared to that of the control strain. There was a negative correlation between *OBP28* gene expression and mortality in adult mosquitoes after exposure to deltamethrin.

**Conclusions:**

To our knowledge, this study shows for the first time a correlation between the expression of a gene coding for OBP and insecticide resistance in mosquitoes. The potential resistance mechanism that was elucidated provides a new target gene for the surveillance of resistance in mosquitoes.

**Graphical abstract:**

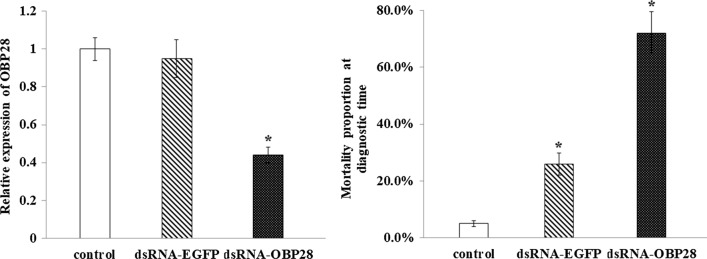

## Abstract (article highlights)

1. RNA sequencing showed that* OBP28* gene expression was higher in the deltamethrin-resistant strains.
2. The qPCR results verified that *OBP28* gene expression was higher in the resistant strains.
3. RNA interference silenced the *OBP28* gene at an efficiency of more than 50%.
4. The sensitivity of *Cx. quinquefasciatus* to deltamethrin increased after *OBP28* had been silenced.

## Background

*Culex quinquefasciatus* is a major vector of Bancroft’s filariasis in China, and an important vector of Japanese encephalitis and other infectious diseases such as West Nile virus and western equine encephalomyelitis [1, 2]. Although chemical control can be effective for the rapid control of mosquitoes [3], the development of resistance among mosquitoes is increasing with the long-term and intensive use of insecticides, and has become a major obstacle in the control of mosquito-borne diseases [4]. The larvae of *Cx. quinquefasciatus* inhabit eutrophic waters with a high organic content, and may come into contact with various types of insecticides as a consequence. In many provinces and cities of southern China, *Cx. quinquefasciatus* already exhibits different degrees of resistance to pyrethroid insecticides. In certain regions of a large number of provinces, such as Shandong, Hunan and Guangdong, resistance levels have increased by a factor of one hundred to several thousands [5–7]. Understanding the mechanisms of resistance is essential for formulating reasonable chemical control strategies. The discovery and further investigation of resistance-related genes in *Cx. quinquefasciatus* should help scientists to develop new methods and measures for the detection and management of resistance.

The olfactory system of insects plays a very important role in their survival and propagation [8], and is an important research area for the development of new control strategies designed to prevent mosquito-borne infections [9]. In the chemosensory systems of insects, olfactory function is primarily mediated by soluble binding proteins such as odorant-binding proteins (OBPs). These proteins are mainly present in the lymph of insect olfactory organs [10]. OBPs transfer odor substances through the sensillum lymph to olfactory receptors, and thus promote the sensitivity of olfactory organs [11]. The interaction between odorant molecules and OBPs may be a first step in olfactory molecule recognition and signal transduction in insects [12, 13]. In *Helicoverpa armigera*, OBP10 interacts with the insect repellent 1-dodecene [14]. Mao et al. [15] found that *Cx. quinquefasciatus* OBP1 shows strong affinity for (5R,6S)-6-acetoxy-5-hexadecanolide under conditions of high pH, and therefore considered that OBP1 might be of use as a potential molecular target of oviposition attractants in mosquitoes. Honeybee OBP ASP2 specifically interacts with imidacloprid, a neonicotinoid insecticide, which affects olfaction and cognition [16]. Xiong et al. [17] showed that upregulation of OBP protein C01 expression in *Tribolium castaneum* resulted in reduced sensitivity to dichlorvos and carbofuran. An increase in affinity between OBPs and exogenous insecticides is one of the major mechanisms in the development of insecticide resistance in insects. The* OBP9* gene of *Spodoptera litura* was significantly upregulated after treatment with chlorpyrifos, and mortality increased significantly when this gene was deleted [18]. These results indicate that the toxicity of insecticides may be reduced by their interaction with OBPs. Thus, this mechanism may play a role in insect resistance to insecticides [19].

In recent years, with the rapid development of RNA interference (RNAi) technology, double-stranded RNA (dsRNA)-mediated gene silencing has been increasingly applied to study gene functions [20]. For example, RNAi technology has been used to confirm that the* PcE1*,* PcE7* and* PcE9* genes are involved in the detoxification process of fenpropathrin in *Panonychus citri* [21]. By using RNAi, Hu et al. [22] showed that the* prosalpha6* gene correlated with stress responses to deltamethrin in *Drosophila melanogaster*. Another study showed that* CYP9J40* and* CYP6AA7* gene overexpression was associated with the resistance of *Cx. quinquefasciatus* to deltamethrin [23]. Liu et al. [24] cloned the permethrin resistance-associated opsin gene of *Culex pipiens pallens* and compared its homology with other insect resistance-associated genes, and hypothesized that this gene participates in the resistance mechanism of *Cx. pipiens pallens* to permethrin.

A high-throughput sequencing study showed that* OBPjj7a*, and *OBP28* in particular, of *Cx. quinquefasciatus* may be involved in the development of resistance [25]. In the present study, a series of bioassays were conducted to determine the relationship between* OBP28* and the resistance of *Cx. quinquefasciatus* to deltamethrin. Bioassays were conducted on both larvae and adults of three strains: a susceptible strain (SS) and two resistant strains [deltamethrin-resistant strain 1 (HN) and deltamethrin-resistant strain 2 (RR)]. The HN strain was tested directly. The RR strain was tested directly and with silencing of* OBP28* and *egfp*, the gene that codes for enhanced green fluorescent protein (EGFP). Changes in the expression of *OBP28* and deltamethrin sensitivity were examined after gene silencing. Finally, a correlation analysis was performed between* OBP28* gene expression in the seven different *Cx. quinquefasciatus* strains examined [three laboratory strains and four field-collected strains (henceforth ‘field strains’)] and their levels of resistance to deltamethrin.

## Methods

### Mosquito strains

Three laboratory strains of *Cx. quinquefasciatus* were used in this study. SS is a laboratory strain originally from Guangzhou that has been kept in the laboratory for more than 10 years without exposure to any insecticides. The HN strain was collected from Haikou city, Hainan province in 2013 and was reared in the laboratory until use in this study. The RR strain was obtained by exposing the HN strain to deltamethrin in the laboratory for 30 generations. The three strains have been kept in the same laboratory under the same feeding conditions. Field strains of *Cx. quinquefasciatus* were collected in four regions of Guangxi province and Hainan province, China: Beihai, Guangxi (BH; 21°28′57″N, 109°8′4″E); Liuzhou, Guangxi (LZ; 24°19′59″N, 109°26′38″E); Sanya, Hainan [SY1 (18°25′7″ N, 109°53′15″ E) and SY2 (18°15′14″N, 109°31′20″E)]. Pipettes and mesh screens were used to obtain the field strains of *Cx. quinquefasciatus* from sewage stored in containers and from underground sewage systems. The larvae and adults were bioassayed after the field strains had been reared for one generation in the laboratory. The mosquitoes were reared at 26 ± 1 °C, 75 ± 5% relative humidity, and under a light:dark schedule (L:D) of 14 h:10 h. The adult mosquitoes of seven strains were fed with 8% sugar water for 3–5 days after emergence, then fed with blood meals to breed the next generation.

### Insecticide resistance bioassays

The laboratory-susceptible strain, SS, and the two deltamethrin-resistant strains, HN and RR, were used for the insecticide resistance bioassays. Bioassays were performed on the larvae and adults of each strain of* Cx. quinquefasciatus* to determine the median lethal concentration (LC_50_) [26, 27] of deltamethrin for each.

For the larval bioassays, serial dilutions of deltamethrin (five to seven concentrations) were prepared using acetone and deionized water; acetone was used as the control. Each concentration was tested on 30 larvae that were between the end of the third instar and the beginning of the fourth instar; there were three repeats for each concentration. Mortality was recorded after 24 h. The experiments were performed at 25 °C, 75% relative humidity and under a L:D of 14 h:10 h.

The LC_50_ values were calculated using Schoofs and Willhite’s [28] probit analysis program. The resistance ratio [29] is the ratio of the estimated LC_50_ of each strain to the LC_50_ of the susceptible strain (SS). When the resistance ratio is < 5, the field population is considered susceptible; when the resistance ratio is between 5 and 10, the mosquitoes are considered to have moderate resistance; and when the resistance ratio is > 10, the mosquitoes are considered highly resistant.

Adult female *Cx. quinquefasciatus* were collected at 3–5 days after emergence, and bioassays were performed using the Centers for Disease Control and Prevention (CDC) bottle bioassay [31, 32]. Deltamethrin was diluted in acetone to prepare a 5 µg/ml stock solution. A 1-ml volume of this solution was evenly spread over the inside of each 250-ml glass bottle. The bottles were rotated to allow the insecticidal agent to spread evenly over the inside of the bottles, and the bottles were then placed in a drawer in the dark for no more than 24 h. Twenty female mosquitoes were placed in each treatment bottle, and each treatment was repeated three times. The criteria for death were inability to stand or fly. The mortality of the female mosquitoes after 24 h of exposure was used as the standard for resistance [32]. All the experiments were performed at 25 °C, 75% relative humidity and a L:D of 14 h:10 h. The experimental groups were treated with the deltamethrin and acetone mixture, and the control group with the acetone solution only.

According to the standard developed by the World Health Organization (based on the recommendations of CDC) [30, 32], the resistance levels of adult mosquitoes were classified as follows: 98–100% mortality denoting sensitivity, 80–97% mortality denoting possible resistance, and mortality lower than 80% denoting resistance.

### RNA extraction, complementary DNA synthesis and real-time quantitative polymerase chain reaction

Total RNA from the three laboratory strains of *Cx. quinquefasciatus* was extracted using a method employing TRIzol. For each strain, there were 20 female mosquitoes per reaction tube and three replicate tubes. After the concentration and optical density (260/280 nm and 260/230 nm) of the RNAs had been measured, the RNA fragments were examined using an Agilent 2100 Bioanalyzer to ensure that they were of sufficient quality. One microgram of messenger RNA in a 20-µl reaction volume was reverse transcribed into complementary DNA (cDNA) in accordance with the Prime Script RT Reagent Kit with genomic DNA Eraser protocol under the following conditions: 37 °C for 15 min, 85 °C for 5 s, followed by a 4 °C hold. Ribosomal protein L8 gene [33] and 18S ribosomal RNA gene [26] were used as the internal control genes for calibration. The primers and probes used for the target and internal control genes were synthesized based on the sequences given in Table 1. cDNA was used as the template for the real-time quantitative polymerase chain reaction (qPCR) using the Premix Ex Taq qPCR Reagent Kit protocol; for the negative control, double-distilled H_2_O was used in place of the template. The qPCR system included 10 µl of Premix Ex Taq (probe-based qPCR), 0.4 µl of PCR forward primer, 0.4 µl of PCR reverse primer, 0.8 µl of probe, 2 µl of DNA template and 6.4 µl of sterile water. The reaction conditions were as follows: 40 cycles of denaturation at 95 °C for 30 s, amplification at 95 °C for 5 s then at 60 °C for 30 s, and final extension at 50 °C for 30 s. There were three technical repeats and three biological repeats per reaction.

**Table 1:** Primers and probes used for the real-time quantitative polymerase chain reaction

Primer description	Primer name	Primer sequence
18S ribosomal RNA (rRNA)	18S rRNA F	5′ATTACGTCCCTGCCCTTTGTAC3′
	18S rRNA R	5′CACCTTCAAAGACCTCACTAAATAATCC3′
	18S rRNA P	5′CACCGCCCGTCGCTACTACCGA3′
Ribosomal protein L8 (RPL8)	RPL8 F	5′AGTTCAAGCTCCGCAAGCA3′
	RPL8 R	5′CACGAACTGGCCGGTGTAC3′
	RPL8 P	5′TTCATCGCCGCCGAGGGC3′
Odorant-binding protein 28 (OBP28)	OBP28 F	5′CGAAGATGAATCGCTTGCAA3′
	OBP28 R	5′TGGCCTGCTCTTTGTCTGAA3′
	OBP28 P	5′TTCGTACTTGCACACGTTCAGGGCC3′

### dsRNA synthesis and RNAi

The coding sequence of the* OBP28* gene was retrieved from the National Center for Biotechnology Information database, and dsRNA primers were designed using E-RNAi online. The primers were synthesized according to the following sequences: OBP-T7F, taatacgactcactatagggGCGTTGTTTTTGACGGTTTT; and OBP-T7R, taatacgactcactatagggTCTTCTCACCGATCCACCTT. The full-length dsRNA target was the 440 base pairs (bp) located between nucleotides 56 and 495 of the target gene. A 287 bp EGFP was used as the negative control (EGFP-T7F, taatacgactcactatagggCAGTGCTTCAGCCGCTAC; and EGFP-T7R, taatacgactcactatagggGTTCACCTTGATGCCGTTC).

The following reverse transcription-polymerase chain reaction system (50 µl) was prepared: 5 µl of 10× LA buffer, 8 µl of dNTPs (2.5 mM), 1 µl of forward primer (10 µM), 1 µl of reverse primer (10 µM), 2 µl of cDNA template, 0.5 µl of LA Taq (5 U/µl), and 32.5 µl of double-distilled H_2_O. cDNA synthesized according to the above method and the EGFP plasmid were used as templates for PCR amplification. The PCR products were subjected to agarose gel electrophoresis and sequencing analysis to confirm the target bands. The PCR products were purified using a TaKaRa MiniBest DNA Fragment Purification Kit to obtain high-purity DNA for subsequent in vitro transcription. A 20-µl transcription reaction system was prepared according to the TaKaRa MEGA script RNAi In Vitro Transcription Reagent Kit protocol as follows: 2 µl of 10× T7 reaction buffer, 2 µl of ATP/CTP/UTP/GTP, 2 µl of T7 enzyme mix, and 1 µg of DNA template; nuclease-free water was added to a total volume of 20 µl. The reaction was carried out at 37 °C overnight. Pure dsRNA was obtained after nuclease digestion and dsRNA purification. The dsRNA concentration was measured using a NanoDrop spectrophotometer and subsequently diluted to 600 ng/µl.

Female *Cx. quinquefasciatus* of the RR strain were collected on days 3–5 after emergence and fasted for 4 h before being anesthetized with CO_2_. The dsRNA-OBP28 was microinjected at 0.5 µl/mosquito, after which the mosquitoes were placed in new mosquito cages and fed 8% sugar water. Control groups were set up simultaneously, one with an injection of dsRNA-EGFP and the other with no treatment. Samples were collected at 24 h, 48 h and 72 h after microinjection. There were three repeats for each treatment, and each repeat comprised 20 female mosquitoes. The expression level of* OBP28* was determined by qPCR.

### Determination of deltamethrin sensitivity in *Cx. quinquefasciatus* after RNAi

Bioassays were performed on mosquitoes after RNAi according to the steps described above, and sensitivity to deltamethrin determined for the experimental and control groups.

### Functional validation using field strains of *Cx. quinquefasciatus*

The sensitivity of the field strains to deltamethrin was determined and the expression levels of the* OBP28* gene measured using the methods described above.

### Statistical analysis

All the statistical analyses were conducted using SPSS 21.0 software (IBM, Chicago, IL). The deltamethrin bioassay results for the three laboratory strains and four field strains (mortality after deltamethrin treatment) were analyzed with Fisher’s exact test. The 2^−ΔΔCT^ method was employed to calculate the relative level of* OBP28* gene expression, as well as the level of expression after interference. LC_50_ values for the insecticide were calculated by log concentration-probit (mortality) regression [34]. Student’s *t*-test was used, and *P* > 0.05 was considered statistically non-significant.

## Results

### Deltamethrin resistance levels in the three laboratory strains of *Cx. quinquefasciatus*

**Fig. 1:**
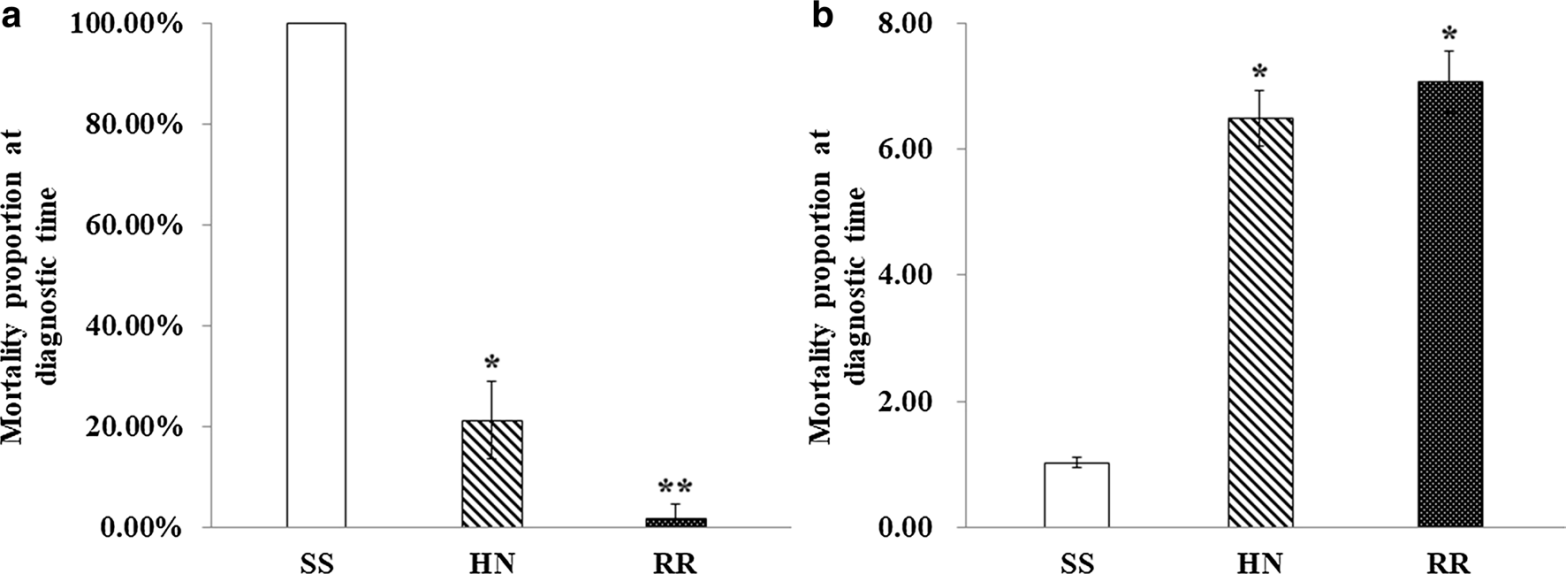
**a**, **b** Resistance to deltamethrin and relative expression of the gene coding for odorant-binding protein 28 (*OBP28*) in adult mosquitoes of three laboratory strains [susceptible strain (*SS*), deltamethrin-resistant strain 1 (*HN*), and deltamethrin-resistant strain 2 (*RR*)]. **a** Sensitivity of strains SS, HN and RR to deltamethrin determined using a distinguishing dosage method. **b** Relative expression of* OBP28* in strains SS, HN and RR determined by real-time quantitative polymerase chain reaction (qPCR). * *P* < 0.05, ** *P* < 0.01

The LC_50_ values for larvae of the SS, HN and RR strains were 0.0000029 µg/ml, 0.014 µg/ml and 0.572 µg/ml, respectively [35]. The two resistant strains both displayed high resistance levels (Table 2). The average mortality of adult mosquitoes was 100.0 ± 0.0%, 21.33 ± 7.64% and 1.67 ± 2.89%, respectively, for strains SS, HN and RR in the bioassay. The average mortality of strain HN strain was 78.67% lower than that of SS, and the difference was statistically significant (*P* < 0.01). The average mortality of the RR strain was 98.33% lower than that of the SS strain, and the difference was significantly significant (*P* < 0.01). Although the average mortality of the RR strain was 19.66% lower than that of the HN strain, the difference was not significant (*P* > 0.05). Because the mortality of adults of both the HN and RR strains was lower than 80%, adults of both strains were considered resistant and thus could be used for the subsequent resistance experiments (Fig. 1a).

**Table 2:** Resistance to deltamethrin of larvae of three laboratory strains [susceptible strain (*SS*), deltamethrin-resistant strain 1 (*HN*) and deltamethrin-resistant strain 2 (*RR*)] and four field-collected strains [from Beihai, Guangxi (*BH*); Liuzhou, Guangxi (*LZ*); and Sanya, Hainan (*SY1* and* SY2*)] of *Culex quinquefasciatus* determined by bioassay

Strain	Slope ± SE	LC_50_ (μg/ml)	95% CI	χ2 (*df*)	*Ρ*	Resistance ratio
SS	2.452 ± 0.248	0.0000029	0.00000192–0.00000687	9.265(3)	0.026	1
HN	0.827 ± 0.177	0.014	0.002–0.032	2.521(3)	0.472	4828
RR	0.900 ± 0.116	0.572	0.381–0.782	5.224(4)	0.265	197,241
LZ	1.796 ± 0.211	0.159	0.125–0.195	0.234(3)	0.972	54,828
BH	2.525 ± 0.274	0.130	0.108–0.153	2.681(3)	0.444	44,828
SY2	2.322 ± 0.263	0.083	0.066–0.099	0.714(3)	0.870	28,621
SY1	2.024 ± 0.240	0.101	0.078–0.124	0.263(3)	0.967	34,828

*LC*_50_ Median lethal concentration, CI confidence interval

### Expression levels of *OBP28* in the three laboratory strains of *Cx. quinquefasciatus*

Relative levels of* OBP28* gene expression in the three laboratory strains of *Cx. quinquefasciatus* were analyzed by qPCR. The relative expression levels in strains SS, HN and RR were 1.03 ± 0.08, 6.49 ± 0.45 and 7.07 ± 0.49, respectively. Compared to the SS strain,* OBP28* gene expression in the two resistant strains was upregulated; the relative expression level in the HN strain was 6.30-fold higher than that in strain SS (*P* < 0.01); the relative expression level in strain RR was 6.86-fold higher than that in strain SS (*P* < 0.05). Levels of expression in both resistant strains were significantly different from that of the SS strain; the levels of expression of the two resistant strains were not significantly different (*P* > 0.05) (Fig. 1b).

### Confirmation of the most appropriate duration of RNAi after dsRNA microinjection of strain RR

**Fig. 2:**
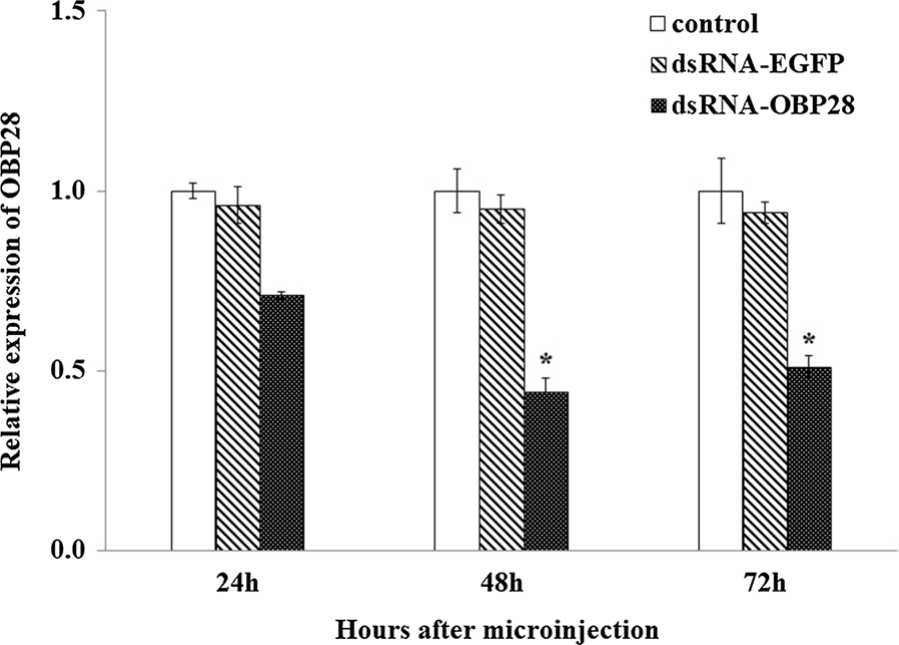
Relative expression levels of *OBP28* at 24 h, 48 h and 72 h after microinjection of double-stranded RNA (*dsRNA*) into laboratory strain RR. The interference effects of dsRNA microinjection were compared for the same time periods. Each column indicates the mean expression level. * *P* < 0.05.* EGFP* Enhanced green fluorescent protein; for other abbreviations, see Fig. 1

Expression levels of *OBP28* after different durations of RNAi were detected using qPCR (Fig. 2). After 24 h, 48 h and 72 h, the expression levels of* OBP28* in the experimental groups decreased compared to those in the control group. Student’s *t*-test showed that the decrease in* OBP28* expression was not significantly different between the groups after 24 h (*P* > 0.05). However, after 48 h and 72 h, there was a significant decrease in* OBP28* expression in the dsRNA-OBP28 group (*P* < 0.05). Compared to the dsRNA-EGFP group,* OBP28* expression in the dsRNA-OBP28 group decreased by 26.04%, 53.68% and 45.74% at 24 h, 48 h and 72 h, respectively. Based on the above results, the subsequent experiments were conducted at 48 h after dsRNA injection.

### Interference effect of microinjection with dsRNA on *OBP28* and changes in deltamethrin sensitivity in strain RR

**Fig. 3:**
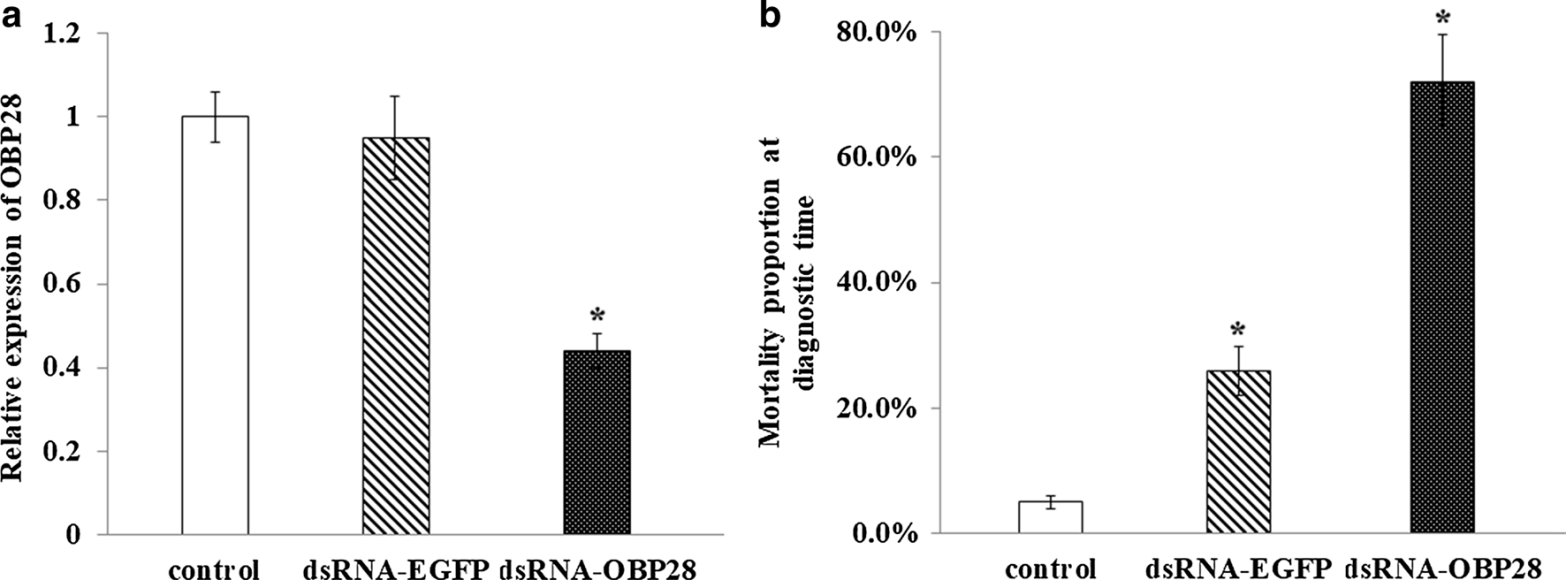
**a**, **b** Resistance to deltamethrin and relative expression of* OBP28* in laboratory strain RR. **a** Relative expression of* OBP28* in strain RR 48 h after microinjection with dsRNA-OBP28 and dsRNA-EGFP, as determined by qPCR. The blank control did not undergo any treatment. **b** CDC bottle bioassay to detect resistance to deltamethrin in strain RR 48 h after microinjection with dsRNA-OBP28 and dsRNA-EGFP. The blank control did not undergo any treatment. For abbreviations, see Figs. 1 and 2

The interference effect of dsRNA on* OBP28* gene expression levels in *Cx. quinquefasciatus* at 48 h after microinjection were determined by qPCR. The level of* OBP28* gene expression in strain RR injected with dsRNA-OBP28 was 53.68% lower than that of the control group injected with dsRNA-EGFP, and there was a statistically significant difference between the levels in the experimental groups injected with dsRNA-OBP28 and dsRNA-EGFP (*P* < 0.05) (Fig. 3a). Resistance in female *Cx. quinquefasciatus* at 48 h after interference was measured using a CDC bottle bioassay. Mortality in *Cx. quinquefasciatus* injected with dsRNA-EGFP was 26.32% after exposure to 5 µg/ml deltamethrin for 30 min. However, mortality in the experimental group injected with dsRNA-OBP28 increased to 72.20% after* OBP28* gene silencing, and was significantly different from that of the control group (*P* < 0.05) (Fig. 3b). These results confirmed that interference of the expression of *OBP28* decreased deltamethrin resistance in *Cx. quinquefasciatus*.

### Resistance levels and *OBP28* gene expression levels in the four field strains of *Cx. quinquefasciatus*

**Fig. 4:**
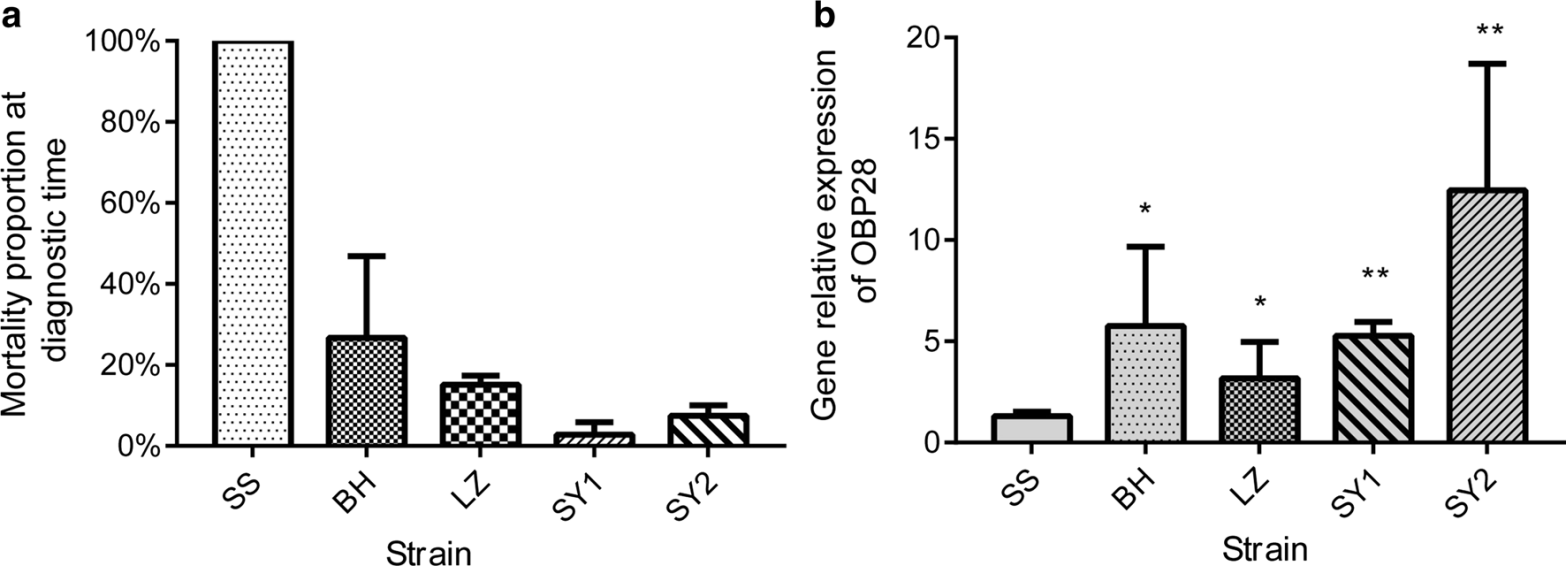
**a**, **b** Resistance to deltamethrin and relative expression of* OBP28* in the four field-collected strains [from Beihai, Guangxi (*BH*); Liuzhou, Guangxi (*LZ*); and Sanya, Hainan (*SY1* and *SY2*)]. **a** Sensitivity of the four strains to deltamethrin determined by a CDC bottle bioassay. **b** Relative expression of* OBP28* in the four strains determined by qPCR. For other abbreviations, see Fig. 1

Adult mosquitoes were exposed to 5 µg/ml deltamethrin for 30 min in the bioassays. Mortality of the field strains BH, LZ, SY1 and SY2 was 26.69 ± 20.20%, 15.10 ± 2.25%, 2.83 ± 3.09% and 7.42 ± 2.66%, respectively (Fig. 4a). The mortality of each of these four strains, which was lower than 50%, met the resistance standard. *OBP28* gene expression determined by qPCR was upregulated in the field strains compared to the SS strain; the relative levels in strains BH, LZ, SY1 and SY2 were 5.76 ± 3.91, 3.17 ± 1.80, 5.27 ± 0.69 and 12.45 ± 6.26, respectively (Fig. 4b). The levels in the BH and LZ strains were significantly different from that in the SS strain at *P* < 0.05; in the SY1 and SY2 strains, the levels were significantly different from that in the SS strain at *P* < 0.01.

## Discussion

Deltamethrin-resistance mechanisms have been elucidated in various insects, including at an epidermal [36] and metabolic level [37], and due to knockdown resistance [38]. Olfaction plays an important role in the biology of mosquitoes. OBPs are multifunctional proteins that can recognize and transport odorant molecules present in the environment and exchange information with the environment to guide a series of behavioral processes in insects [39]. OBPs can bind pheromones or poisonous odors present in the environment and transport them to the hemolymph for detection by odorant receptors. Some OBPs contain a* N*,*N*-diethyl-3-methylbenzamide binding site, which might be associated with the ability of mosquitoes to avoid that particular compound [40]. Ingham et al. [42] showed that expression of the protein SAP2 increases significantly in pyrethroid-resistant *Anopheles gambiae* and is mainly concentrated in the legs. It was speculated that SAP2 mediates the avoidance of *An. gambiae* to insecticides, which manifests as resistance [41].

The expression of* Obp99a* was found to change significantly in *Drosophila* as a result of oxidative stress after the insect’s exposure to parathion [42]. High-throughput sequencing analyses of genes of 12 strains of mosquitoes that play a role in insecticide resistance showed that *OBP28* might be associated with deltamethrin resistance in mosquitoes [25]. To our knowledge, the present study is the first to use qPCR to confirm that* OBP28* gene expression levels are indeed significantly different between susceptible and resistant strains of *Cx. quinquefasciatus*. Indeed, *OBP28* gene expression levels in deltamethrin-resistant strains were significantly upregulated, and RNAi technology was employed to further investigate the relevant functions of this gene.

At present, RNAi technology is extensively applied in studies on gene functions. Its use does not alter the genome of mosquitoes and thus does not damage their genetic diversity. At present, the introduction of dsRNA into insects is mainly achieved by microinjection or through feeding. In this study, a microinjection method was used to introduce dsRNA into adult *Cx. quinquefasciatus*. The best interference time for RNAi was investigated, and* OBP28* gene expression levels at different time periods determined using 24 h as the time interval unit (interference time). The interference efficiency first increased and then decreased with time, and was highest at 48 h. Therefore, 48 h was selected as the time point of analysis in the subsequent experiments. In *Aedes albopictus*, the expression level of a silenced vitellogenin-2 gene was most significantly reduced at day 3 after dsRNA microinjection and significantly upregulated after day 4 [43]. Singh et al. [44] used a dipping method to interfere with the β-tubulin gene in *Aedes aegypti* and showed that 7 days of interference was required to reach the peak effect. A feeding method was used to silence the topoisomerase inhibitor-suppressed gene in *Anopheles stephensi* [45]; the interference effect was best at 3 h, plateaued at 6 h, and had decreased significantly by 24 h. The best interference time in our study may have been different from the latter’s due to the different dsRNA concentrations, insect stages, interference methods and target genes examined [45]. The results of our RNAi experiments provided further evidence of a negative relationship between* OBP28* gene expression levels and deltamethrin resistance in mosquitoes.

The negative correlation suggests that there may be an interaction between OBP28 and deltamethrin which enables mosquitoes to sense the presence of this insecticide and thus show avoidance responses. Thus, mosquitoes may be able escape the insecticide’s action, and new phenotypes develop as a result of this behavioral resistance. To further analyze the association between the* OBP28* gene and resistance, mosquitoes were collected from four geographic locations in the field. The results indicated that resistance increased in adults due to an increase in* OBP28* expression. Overall, both the experimental results of the RNAi and verification of the results using the four field strains confirmed a significant relationship between* OBP28* gene expression and deltamethrin resistance in mosquitoes.

## Conclusions

This study validates, to our knowledge for the first time, the hypothesized relationship between the level of expression of a gene coding for an OBP and insecticide resistance in mosquitoes, and elucidates a novel insecticide resistance mechanism in mosquitoes. Furthermore, the results suggest that *OBP28* can be used as a new target gene for the surveillance of deltamethrin resistance in *Cx. quinquefasciatus*. In future work, more olfactory gene products, such as OBPs, odorant receptors and gustatory receptors, which interact with different types of insecticides, should be analyzed to identify novel resistance mechanisms in mosquitoes to provide new methods for detecting resistance as well as new targets for mosquito control.

## Data Availability

The datasets supporting the conclusions of this article are included within the article.

## Abbreviations

CDC: Centers for Disease Control and Prevention
cDNA: Complementary DNA
dsRNA: Double-stranded RNA
EGFP: Enhanced green fluorescent protein
HN: Deltamethrin-resistant strain 1
LC_50_: Median lethal concentration
OBP: Odorant-binding protein
qPCR: Real-time quantitative polymerase chain reaction
RNAi: RNA interference
RPL8: Ribosomal protein L8
RR: Deltamethrin-resistant strain 2
SS: Susceptible strain
18S rRNA: 18S ribosomal RNA

## References

[1] Nuss AB, Brown MR, Murty US, Gulia-Nuss M (2018). Insulin receptor knockdown blocks filarial parasite development and alters egg production in the southern house mosquito, *Culex quinquefasciatus*. PLoS Negl Trop Dis.

[2] Xu N, Sun XH, Liu ZH, Xu Y, Sun Y, Zhou D (2019). Identification and classification of differentially expressed genes in pyrethroid-resistant *Culex pipiens pallens*. Mol Genet Genom.

[3] Ding YR, Yan ZT, Si FL, Li XD, Mao QM, Asghar S (2020). Mitochondrial genes associated with pyrethroid resistance revealed by mitochondrial genome and transcriptome analyses in the malaria vector *Anopheles sinensis* (Diptera: Culicidae). Pest Manage Sci.

[4] Reid WR, Zhang L, Gong Y, Li T, Liu N (2018). Gene expression profiles of the southern house mosquito *Culex quinquefasciatus* during exposure to permethrin. Insect Sci.

[5] Liu HM, Yang PP, Cheng P, Wang HF, Liu LJ, Huang XD (2015). Resistance level of mosquito species (Diptera: Culicidae) from Shandong province, China. Int J Insect Sci.

[6] Huang J, Huang Y, Yao SY, Shou ZH, Zhou XF (2014). Determination of the resistance of *Culex pipiens* to four kinds of insecticides in Zhuzhou city. Pract Prev Med.

[7] Li CL (2015). Study on the resistance of *Culex quinquefolium* larvae to common insecticides in Guangzhou. Chin J Hyg Insect Equip.

[8] Leal WS (2012). An acute olfactory system is essential for reproduction-the raison d'etre for adult insects. Proc Am Philos Soc.

[9] Paramasivan R, Sivaperumal R, Dhananjeyan KJ, Thenmozhi V, Tyagi BK (2007). Prediction of 3-dimensional structure of salivary odorant-binding protein-2 of the mosquito *Culex quinquefasciatus*, the vector of human lymphatic filariasis. In Silico Biol.

[10] Pelosi P, Zhou JJ, Ban LP, Calvello M (2006). Soluble proteins in insect chemical communication. Cell Mol Life Sci.

[11] Leal WS (2013). Odorant reception in insects: roles of receptors, binding proteins, and degrading enzymes. Annu Rev Entomol.

[12] Smith DP (2007). Odor and pheromone detection in *Drosophila melanogaster*. Pflugers Arch.

[13] Leal WS, Chen AM, Erickson ML (2005). Selective and pH-dependent binding of a moth pheromone to a pheromone-binding protein. J Chem Ecol.

[14] Sun YL, Huang LQ, Pelosi P, Wang CZ (2012). Expression in antennae and reproductive organs suggests a dual role of an odorant-binding protein in two sibling *Helicoverpa* species. PLoS ONE.

[15] Mao Y, Xu X, Xu W, Ishida Y, Leal WS, Ames JB (2010). Crystal and solution structures of an odorant-binding protein from the southern house mosquito complexed with an oviposition pheromone. Proc Natl Acad Sci USA.

[16] Li H, Wu F, Zhao L, Tan J, Jiang H, Hu F (2015). Neonicotinoid insecticide interact with honeybee odorant-binding protein: implication for olfactory dysfunction. Int J Biol Macromol.

[17] Xiong W, Gao S, Lu Y, Wei L, Mao J, Xie J (2019). Latrophilin participates in insecticide susceptibility through positively regulating *CSP10* and partially compensated by *OBPC01* in *Tribolium castaneum*. Pestic Biochem Physiol.

[18] Lin X, Jiang Y, Zhang L, Cai Y (2018). Effects of insecticides chlorpyrifos, emamectin benzoate and fipronil on *Spodoptera litura* might be mediated by OBPs and CSPs. Bull Entomol Res.

[19] Pelosi P, Iovinella I, Zhu J, Wang G, Dani FR (2018). Beyond chemoreception: diverse tasks of soluble olfactory proteins in insects. Biol Rev Camb Philos Soc.

[20] Kalinna BH, Brindley PJ (2007). Manipulating the manipulators: advances in parasitic helminth transgenesis and RNAi. Trends Parasitol.

[21] Leaute-Labreze C, Hoeger P, Mazereeuw-Hautier J, Guibaud L, Baselga E, Posiunas G (2015). A randomized, controlled trial of oral propranolol in infantile hemangioma. N Engl J Med.

[22] Hu J, Xu Q, Chi Q, Liu W, Li F, Cheng L (2016). Identification of proteasome alpha6 subunit associated with deltamethrin resistance in *Drosophila melanogaster* kc cells. Arch Insect Biochem Physiol.

[23] Delannay C, Goindin D, Kellaou K, Ramdini C, Gustave J, Vega-Rua A (2018). Multiple insecticide resistance in *Culex quinquefasciatus* populations from Guadeloupe (French West Indies) and associated mechanisms. PLoS ONE.

[24] Liu HQ, Liu YB, Song YP, Duan XL, Cheng NN (2010). Cloning and bioinformatics analysis of full length cDNA of permethrin-resistance associated opsin gene PR-OP of *Culex pipens pallens*. J Northwest A&F Univ (Nat Sci Ed).

[25] Liu QM, Li CX, Wu Q, Shi QM, Sun AJ, Zhang HD (2017). Identification of differentially expressed genes in deltamethrin-resistant *Culex pipiens quinquefasciatus*. J Am Mosq Control Assoc.

[26] Li T, Liu L, Zhang L, Liu N (2014). Role of G-protein-coupled receptor-related genes in insecticide resistance of the mosquito, *Culex quinquefasciatus*. Sci Rep.

[27] Li T, Liu NN (2010). Inheritance of permethrin resistance in *Culex quinquefasciatus*. J Med Entomol.

[28] Schoofs GM, Willhite CC (1984). A probit analysis program for the personal computer. J Appl Toxicol.

[29] Orshan L, Kelbert M, Pener H (2005). Patterns of insecticide resistance in larval *Culex pipiens* populations in Israel: dynamics and trends. J Vector Ecol.

[30] Centers for Disease Control and Prevention. Parasites—CDC bottle bioassay. 2013. https://www.cdc.gov/malaria/resources/pdf/fsp/ir_manual/ir_cdc_bioassay_en.pdf.

[31] Richards SL, Balanay JAG, White AV, Hope J, Vandock K, Byrd BD (2018). Insecticide susceptibility screening against *Culex* and *Aedes* (Diptera: Culicidae) mosquitoes from the United States. J Med Entomol.

[32] Test procedures for insecticide resistance monitoring in malaria vector mosquitoes. 2016. https://www.who.int/en//iris/bitstream/handle/10665/250677/9789241511575-eng.pdf.

[33] Wu Q, Li CX, Liu QM, Guo XX, Shi QM, Zhang HD (2020). RNA interference of odorant receptor CquiOR114/117 affects blood-feeding behavior in *Culex quinquefasciatus*. Acta Trop.

[34] Finney DJ (1971). Probit analysis.

[35] Shen RX, Wang YT, Chun X, Wu JH, Zhao TY, Chen Y (2020). Analysis of deltamethrin resistance-related genes based on the transcriptome of *Culex pipiens quinquefasciatus*. Parasitol Parasit Dis.

[36] Togawa T, Augustine Dunn W, Emmons AC, Willis JH (2007). CPF and CPFL, two related gene families encoding cuticular proteins of *Anopheles gambiae* and other insects. Insect Biochem Mol Biol.

[37] Dang K, Doggett SL, Veera Singham G, Lee CY (2017). Insecticide resistance and resistance mechanisms in bed bugs, *Cimex* spp. (Hemiptera: Cimicidae). Parasit Vectors.

[38] Soderlund DM, Knipple DC (2003). The molecular biology of knockdown resistance to pyrethroid insecticides. Insect Biochem Mol Biol.

[39] Ren ZZ, Liu XY, Hu MY (2010). Structure and function of olfactory related proteins in insects. Chin J Biochem Mol Biol.

[40] Hallem EA, Dahanukar A, Carlson JR (2006). Insect odor and taste receptors. Annu Rev Entomol.

[41] Ingham VA, Anthousi A, Douris V, Harding NJ, Lycett G, Morris M (2020). A sensory appendage protein protects malaria vectors from pyrethroids. Nature.

[42] Li N, Chen Z, Liu W, Chi QP, Hu JL, Cheng LG (2015). RNA-Seq (quantitative) analysis of the stress gene in* Drosophila* cells treated with parathion. Chin J Appl Entomol.

[43] Dittmer J, Alafndi A, Gabrieli P (2019). Fat body-specific vitellogenin expression regulates host-seeking behaviour in the mosquito *Aedes albopictus*. PLoS Biol.

[44] Singh AD, Wong S, Ryan CP, Whyard S (2013). Oral delivery of double-stranded RNA in larvae of the yellow fever mosquito, *Aedes aegypti*: implications for pest mosquito control. J Insect Sci.

[45] Negri A, Ferrari M, Nodari R, Coppa E, Mastrantonio V, Zanzani S (2019). Gene silencing through RNAi and antisense Vivo-Morpholino increases the efficacy of pyrethroids on larvae of *Anopheles stephensi*. Malar J.

